# 1436. Effect of Personal Protective Equipment Conservation Due to COVID-19 on Multidrug Resistant Organisms in a Hospital Group

**DOI:** 10.1093/ofid/ofad500.1273

**Published:** 2023-11-27

**Authors:** Josephine Fox, Carole Leone, Heather Gasama, Lydia J Grimes- Jenkins, Munigala Satish, Helen Wood, Kenneth Whalen, Jonas Marschall, David K Warren

**Affiliations:** Barnes-Jewish Hospital, Saint Louis, Missouri; BJC Healthcare, Saint Louis, Missouri; Barnes-Jewish Hospital, Saint Louis, Missouri; Barnes-Jewish Hospital, Saint Louis, Missouri; Washington University in St. Louis, St. Louis, MO; Barnes-Jewish Hospital, Saint Louis, Missouri; BJC Healthcare, Saint Louis, Missouri; Washington University School of Medicine in St. Louis, Saint Louis, Missouri; Washington University School of Medicine in St. Louis, Saint Louis, Missouri

## Abstract

**Background:**

Early in the COVID-19 pandemic many healthcare facilities experienced shortages of personal protective equipment (PPE), which required them to conserve and prioritize PPE. We wanted to determine the effect a gown conservation strategy had on nosocomial methicillin-resistant *Staphylococcus aureus* (MRSA), *Clostridioides difficile (*C.diff.) and infections with vancomycin-resistant enterococci (VRE).

**Methods:**

Prior to the pandemic, isolation gowns were required for patients colonized or infected with multidrug-resistant organisms (MDROs) at BJC Healthcare, a 15-site hospital group with >3,000 acute care beds. In April 2020, we initiated a modified contact precautions (CP) strategy for acute care patients colonized with MDROs (identified via surveillance cultures or based on past MDRO infection). Healthcare workers were instructed to wear gloves and follow standard precautions when seeing patients on modified CP. Patients with active MDRO infection and C.diff. were excluded from modified CP. Rates of NHSN laboratory identified (LabID) MRSA bacteremia and C.diff., and nosocomial VRE from blood/urine cultures, were compared before and after the modified CP. The study period was divided into baseline CP (1/01/2019- 8/30/2019) and modified CP (1/01/2021-8/30/2021) periods.

**Results:**

There was no difference in LabID MRSA bacteremia or LabID C.diff. rates in the baseline CP versus the modified CP period (MRSA: 0.069 baseline vs 0.062 modified CP per 1000 pt days, p=0.64, and C.diff.: 3.96 baseline vs 3.66 modified CP per 10,000 pt days, p=0.45), respectively. There was no difference in the nosocomial VRE bacteremia & bacteriuria combined rate (0.097 baseline vs 0.116 per 1000 pt days modified CP, p=0.37). There was no difference in the subsets of nosocomial VRE bacteremia (0.038 baseline vs 0.047 modified CP, p=0.51) or VRE bacteriuria (0.059 baseline vs 0.069 per 1000 pt days modified CP, p=0.54).

**Conclusion:**

We limited gown use to cases of MRSA, VRE and C. difficile infection in a conservation effort, and no longer required it for MRSA or VRE colonization; this change was not associated with increases of MRSA or C.difficile LabID rates nor with nosocomial VRE cases. Our findings highlight the importance of monitoring and evaluating PPE conservation strategies to document their safety.

**Disclosures:**

**All Authors**: No reported disclosures

